# CBD: A Deep-Learning-Based Scheme for Encrypted Traffic Classification with a General Pre-Training Method

**DOI:** 10.3390/s21248231

**Published:** 2021-12-09

**Authors:** Xinyi Hu, Chunxiang Gu, Yihang Chen, Fushan Wei

**Affiliations:** 1 State Key Laboratory of Mathematical Engineering and Advanced Computing, Zhengzhou 450001, China; gcx5209@126.com (C.G.); cyhpaper@163.com (Y.C.); weifs831020@163.com (F.W.); 2 Henan Key Laboratory of Network Cryptography Technology, Zhengzhou 450001, China

**Keywords:** encrypted traffic classification, deep learning, transfer learning, nature language processing, unlabeled pre-training

## Abstract

With the rapid increase in encrypted traffic in the network environment and the increasing proportion of encrypted traffic, the study of encrypted traffic classification has become increasingly important as a part of traffic analysis. At present, in a closed environment, the classification of encrypted traffic has been fully studied, but these classification models are often only for labeled data and difficult to apply in real environments. To solve these problems, we propose a transferable model called CBD with generalization abilities for encrypted traffic classification in real environments. The overall structure of CBD can be generally described as a of one-dimension CNN and the encoder of Transformer. The model can be pre-trained with unlabeled data to understand the basic characteristics of encrypted traffic data, and be transferred to other datasets to complete the classification of encrypted traffic from the packet level and the flow level. The performance of the proposed model was evaluated on a public dataset. The results showed that the performance of the CBD model was better than the baseline methods, and the pre-training method can improve the classification ability of the model.

## 1. Introduction

In recent years, with the rapid development of network technology and people’s gradual awareness of private data, a variety of encryption technologies have been widely used in network communications, resulting in a rapid increase in network encrypted traffic. At the same time, encrypted traffic is also used by some people as the tool to hide activities, which also provides an opportunity for malicious network attackers to hide their command-and control activities. Therefore, encrypted traffic classification can better monitor abnormal conditions in the network, and detect network attack behaviors in time. It has also contributed to the improvement in network service performance and the creation of a good network environment, which has gained widespread attetion from researchers.

Network traffic classification refers to using algorithms to construct a classification model for neiwork traffic, which has three levels of granularity for tasks: sequence-based, packet-based, and flow-based classification. The granularity can be determined according to practical scenarios, such as application classification, protocol identification, service analysis, etc. Network traffic classification plays a significant role in network management, traffic control, and security detection. Meanwhile, the efficient and accurate classification of network traffic is an important foundation for maintaining network security.

However, most of the existing research results are only implemented in a closed environment. The experimental data are often labeled data, and training data and test data often have high similarities. Moreover, some results tend to require more computing resources and time. In a real environment, the data are often unlabeled, which can greatly decrease the performance of many classification models. To solve this problem, researchers have tried to develop a model with a strong generalization ability.

In this work, we propose a model called CBD (based on a convolutional neural network, bidirectional encoder representation from transformers and dense network) that supports unlabeled data and has a certain generalization ability. It can directly learn from unlabeled data and use this knowledge for model training. Model transfer can also be carried out for different datasets, which only need to fine-tune the parameters to complete the goal of encrypted traffic classification. The contributions of this paper are summarized as follows:

- A novel encrypted traffic classification model called CBD is designed. It combines a Convolutional Neural Network (CNN) and Bidirectional Encoder Representation from Transformers (BERT) to automatically learn the features of traffic data from the packet level and flow level to achieve the encrypted traffic classification for applications.
- A general pre-training method suitable for the field of encrypted traffic analysis is proposed. For unlabeled data, this method proposes two tasks of identifying ciphertext packets and identifying continuous flows, to deepen the model’s understanding and learning of encrypted traffic data.
- The CBD model has achieved good results when encrypting traffic classification. In addition, the performence of the CBD model has obvious advantages compared with other methods.

The rest of the paper is organized as follows. Section 2 summarizes the related work of network traffic analysis. In Section 3, we provide a detailed description of the overall model structure. In Section 4, we introduce the specific details of the experiment, and we evaluate and compare the experimental results. Finally, we conclude this paper in Section 5.

## 2. Related Work

As a multidisciplinary subject, machine learning has three important components: data, features, and algorithms. Traditional machine learning methods use different algorithms to build models and take the features of the raw data as input. After training the models, the expected prediction results can be obtained in the testing phase. In recent years, there has been an increase in research on machine learning, including deep learning and transfer learning. There has also been an increase in research on network traffic classification using these methods. In this section, we briefly introduce some methods for network traffic classification, ranging from deep learning methods to classic transfer learning methods, and then to the applications of Transformer model.

### 2.1. Applications of Deep Learning

For traditional machine learning, the relevant features of the data are not obvious in many cases, and it is necessary to perform feature extraction and feature selection to obtain the features that can show the data. This feature engineering often relies on expert experience, and the quality of the features is critical to the final results. With the development of deep learning, due to its end-to-end model structure, which can effectively reduce the high cost of feature engineering, more and more researchers have chosen to use deep learning in various fields. In 2015, Ref. [1] deep learning was first used in the field of traffic analysis, and a large amount of research subsequently emerged.

In 2017, Höchst et al. [2] proposed a method for unsupervised traffic flow classification using the statistical features of flows and clustering based on neural autoencoders. This method was based on the feature vector of the time interval and used the semi-automatic cluster marking method, which can effectively promote the flow classification independent of the known class of traffic. The evaluation experiment was conducted on real datasets captured within four months. The results showed that for seven different classes of mobile traffic classification, this method can achieve an average accuracy of 80% and an average recall rate of 75%.

In 2018, Li et al. [3] proposed a Byte Segment Neural Network (BSNN) for protocol classification based on Recurrent Neural Networks (RNN). BSNN took network datagram as input. First, the datagram was divided into multiple byte segments. These segments were then fed to an encoder based on RNN. The information extracted by the encoder was combined into a representation vector of the entire datagram. Finally, the softmax function was applied to use this vector to predict the application protocol of the datagram, provoding the classification result. Experiments on the real-world data of different protocols showed that the BSNN classification of five protocols can achieve an average F1-score of about 95.82%, which was better than traditional machine-learning-based methods and packet inspection methods.

In 2019, Marín et al. [4] proposed two methods for malicious network traffic detection based on deep learning, depending on different forms of raw data. The method based on the raw packet combines CNN and Long Short Term Memory (LSTM) network, and the method based on the raw flow uses CNN. The experimental results showed that the performance of the method based on the raw packet was equivalent to that obtained through expert knowledge, while the performance of the method based on the raw flow was better than the performance obtained through expert knowledge. Moreover, when the same input features were used, the deep learning model was better than the Random Forest (RF) model used as the benchmark.

In 2020, Pacheco et al. [5] proposed a framework based on machine learning and deep learning for the classification of heterogeneous Internet traffic in satellite communications. The machine learning method chose rf, and the deep learning methods chose 1d and 2dcnn. This solution combined a classification solution in the satellite architecture for QoS management. By dividing the Internet protocol technology and using the multi-label classification method to process the tunnel connection, the Internet flow can be classified. In addition, the specific feature extraction process of the tunnel connection was designed. The experiment verified the reliability of the simulated Internet network placed on the cloud platform, and also provided guidance for future work on the simulated and real satellite platforms.

In 2020, Aceto et al. [6] combined and summarized some existing deep-learning-based traffic classification methods, and proposed a general framework for mobile traffic classification based on deep learning. To evaluate the framework, the authors conducted experiments on three mobile datasets of human user activity. The results showed that the classification result was better than the machine learning baseline method and the deep learning baseline method.

In 2021, Hu et al. [7] proposed a model CNN LSTM Dense Network (CLD-Net) to classify encrypted network traffic based on CNN and LSTM. This model introduced the strategy of recombing traffic in the data-preprocessing part, which can effectively improve the efficiency of neural network feature learning. In addition, a method for converting the format of the time interval between adjacent packets was proposed, which can fuse time information and payload information into a new packet matrix. The method was validated on the public dataset ISCXVPN2016 [8]. The results showed that the method can classify whether encrypted network flows used a Virtual Private Network (VPN) with 98% accuracy, and accurately classify the specific traffic classes of Facebook and Skype applications with 92.89% accuracy.

### 2.2. Applications of Transfer Learning

Yang et al. [9] summarized the different parts of transfer learning in detail, and provided a definition. Researchers have widely used this in various fields and combined it with other technologies to propose many improvements. The combination of transfer learning and deep learning is a well-known method. After the two are combined, through pre-training and parameter fine-tuning, the high training overhead caused by too many neural network parameters can effectively be reduced.

In 2018, Taheri et al. [10] proposed a botnet detection system based on deep learning. In this system, normal and botnet network traffic data were converted into images, which were classified by DenseNet and transfer learning. The evaluation results on the CTU-13 dataset [11] showed that the use of transfer learning can increase the accuracy from 33.41% to 99.98%. The baseline method chose Support Vector Machine (SVM) and logistic regression, and their accuracy rates were 83.15% and 78.56%, respectively. The system also performed well on internal real-time normal datasets and botnet datasets. The real-time processing ability of the system was also better. During the test, it took 0.004868 milliseconds to process each packet from network traffic data.

In the same year, Sun et al. [12] proposed a traffic classification method based on transfer learning, the so-called Transferred Adaboost (TrAdaBoost). This method used TrAdaBoost to extract labeled traffic data from different network traffic sources, and used the maximum entropy (Maxent) model as the basic classifier. The proposed method realizes the transfer of traffic knowledge from the source domain to the target domain. The experiment used a flow dataset collected at Cambridge University. In the case where the training and test datasets were not exactly the same, this method achieved a higher classification accuracy in the new dataset, while the performance of traffic classification based on traditional machine learning models was significantly reduced.

In 2020, Liu et al. [13] proposed a model based on two-way gated recurrent unit and attention mechanism (BGRUA) to classify and identify Web services running on Hyper Text Transfer Protocol over SecureSocket Layer (HTTPS) connections. The two-way GRU can extract the forward and backward features of the byte sequence in the sessions, and the attention mechanism can assign weights to the features according to their contribution to the classification. The experiment was conducted on three datasets, two of which are publicly available HTTPS datasets, whlie the third was a dataset collected from the backbone of China Science and Technology Network. The results showed that BGRUA was superior to the baseline method in terms of accuracy, precision, recall and F1-score.

In 2021, Hu et al. [14] proposed a transferred CLD-Net (tCLD-Net) model combining transfer learning and deep learning on the basis of [7]. It can be trained with only a small amount of labeled data; that is, it can classify encrypted network traffic with a higher accuracy. It pre-trained a CLD-Net model in the source domain dataset, fixed the parameters of the CNN module and trained and tested it in the target domain dataset. The effectiveness of this method was verified on the public dataset ISCXVPN2016 [8], and the results showed that the performance was significantly improved compared with no pre-training. When the training set only occupied 20% of the target domain, the training time was reduced by two-thirds, and the classification accuracy was increased by more than 4%.

Recently, Wang et al. [15] proposed a new method called adaptive fingerprint identification. This method used adversarial domain adaptation [16,17] in transfer learning to obtain high attack accuracy for a small amount of encrypted traffic. Extensive experimental results on multiple datasets showed that the method can achieve 89% accuracy for a small number of encrypted traffic in a closed world setting, and 99% accuracy and 99% recall in an open world setting. Compared with the recent Triplet Fingerprinting [18], this method was more efficient in terms of pre-training time and more scalable, and its attack performance was better than Triplet Fingerprinting in both closed-world evaluation and open-world evaluation.

### 2.3. Applications of Transformer Model

With the wide application of the Transformer model [19] proposed in 2017 in the field of natural language processing (NLP), researchers began to apply the Transformer model to traffic analysis tasks based on the similarity of the research objects.

In 2020, Bikmukhamedo et al. [20] introduced a network traffic model based on the generated Transformer to generate and classify network traffic. The model used autoregressive training to generate network traffic, and then used Kolmogorov–Smirnov statistics to evaluate the generated traffic, to achieve the goal of classification. Experimental results showed that the generated pre-training had a positive impact on the quality of traffic classification tasks. In the case of using the pre-trained model as the feature extractor of the linear algorithm, the classification performance was close to the RF trained on the raw stream. After training the model, the average macro F1-score of the classifier was 4% better than the classifier of the ensemble class.

In 2021, Wang et al. [21] designed a hybrid neural network (Distributed Denial of Service (DDoS) Traffic Classification (DDosTC) model. The model combined an efficient and scalable Transformer and CNN to detect DDoS attacks on Software Defined Network (SDN), and was tested on the latest dataset CIC-DDoS2019 [22]. The experiment was carried out many times by dividing the dataset, and compared with the latest deep learning detection algorithm applied in the field of distributed denial of service intrusion detection. Experimental results showed that the average Area Under Curve (AUC) of DDosTC is 2.52% higher than the current optimization model. In terms of average accuracy, recall and F1-score, DDosTC was more successful than the current optimization model.

Kozik et al. [23] proposed a core anomaly detection classifier in the same year. It is based on the Transformer Encoder component, followed by a feedforward neural network. A time window embedding solution was also proposed, which can process large amounts of data efficiently and has a low memory footprint. The experiment verified the effectiveness of this method on the dataset Aposemat IoT-23 [24], compared it with other classic machine learning algorithms, and discussed their effectiveness in IoT-related environments. The results showed that the average accuracy, recall and F1-score of this method were significantly better than other baseline methods.

Afterwards, Google proposed a pre-trained language representation model BERT based on Transformer [25] in 2019. It became a hot new method in the fields of NLP, computer vision, etc., producing a large amount of related research. However, there is little research on the use of BERT in traffic classification tasks [26].

In 2021, He et al. [26] introduced the BERT model to traffic classification, and proposed a model Payload Encoding Representation from Transformer (PERT) that uses dynamic word embedding technology to automatically extract traffic features. The unlabeled traffic was used to pre-train the encoding network, and the encoding network learned the context distribution of the traffic. When performing downstream classification tasks, the pre-trained network was reused to enhance the classification results. Through experiments on public encrypted traffic datasets and Android HTTPS traffic captured by the authors, the results showed that this method was significantly better than other baseline methods.

We also conducted research based on BERT and combined with CNN. From the perspective of packet and flow, we propose a general pre-training method and design an encrypted traffic classification model. This paper is only for the payloads of the packets. Compared with some other traffic classification studies, this work does not extract any Transmission Control Protocol/Internet Protocol (CP/IP) header information, 5-tuple information (5-tuple is [source IP, source port, destination IP, destination port, transport protocol]), or other obvious ipacket nformation.

## 3. Model Structure

**Figure 1:**
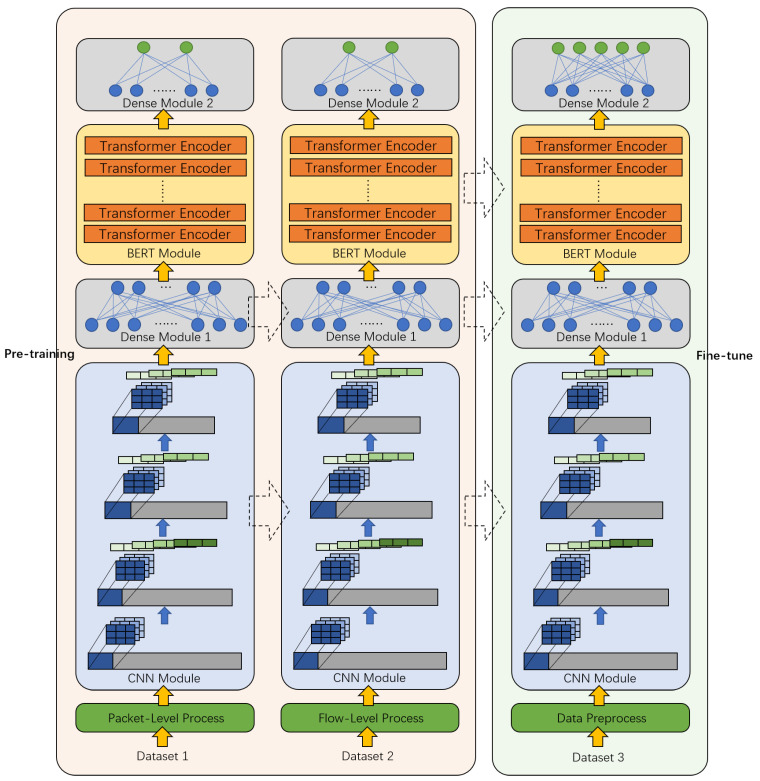
The overall framework of the CBD model.

Encrypted traffic has many characteristics, such as high entropy, unobvious statistical characteristics, and a weak correlation between adjacent bytes. It is difficult to extract features manually and they cannot be well represented. It may be difficult to directly use traditional or classic methods in other fields, such as NLP and CV, to classify encrypted traffic. Therefore, we designed a CBD model for encrypted traffic, and the overall structure is shown in Figure 1, including three modules: CNN module, BERT module, and Dense module. The process of the entire model includes the pre-training process and the fine-tuning process.

The left side of Figure 1 represents the pre-training process, the right side represents the fine-tuning process, and the dotted arrow represents the transfer of the module.

### 3.1. Data Preprocess

For the raw traffic data, the data must be preprocessed before they can be input into the neural network model. The preprocess mainly includes traffic segmentation, traffic cleaning, traffic conversion and time interval integration. Algorithm 1 represents the process.
**Algorithm 1** Preprocessing Algorithm.**Input:** 
Raw network traffic dataset *D*; Number of traffic classes *c*;**Output:** 
Packet stream set *P*;1:**for** each i∈[1,c] **do**2:    Randomly select *n* consecutive 10 packets in *D*;3:    **for** each j∈[1,10×n] **do**4:        Trim ${\mathrm{packet}}_{j}$ and uniform length of 256 bytes, ${\mathrm{packet}}_{j}\to p_{j}^{\prime }$;5:        Generate the packet stream, payload $p_{j}^{\prime }\to p_{j}$;6:        Count the time interval of $p_{j}$ and $p_{j+1}$;7:        **if** the time interval between $p_{j}$ and $p_{j+1}$< 1 sencond **then**8:           Continue9:        **else**10:           Add $p_{0}$ between $p_{j}$ and $p_{j+1}$, where $p_{0}={\left(1,...,1\right)}_{256}$;11:        **end if**12:    **end for**13:    Generate packet stream set $P_{i}=\left\{p_{1},...,p_{0},...,p_{0},...,p_{10*n}\right\}$;14:**end for**15:**return** Packet stream set $P=\{P_{1},P_{2},...,P_{c}\}$;

Line 2 is traffic segmentation. Several flow segments with a window of 10 are randomly intercepted, that is, each flow segment contains 10 consecutive packets.

Line 4 is traffic cleaning. The payload part of each packet is read, and then the length is unified, the first 256 bytes of each packet are intercepted, and 0 is filled in if the result is less than 256 bytes to obtain the raw stream

(1) $$p^{\prime }=\left(b_{1},b_{2},\cdots ,b_{8\times 256}\right).$$

Line 5 is traffic conversion. The raw stream is converted to decimal, and a value of 0-255 is taken, according to each byte; then, a 256-dimensional sequence p is obtained.

Line 6–9 is time interval integration. According to the statistical results of the time interval between two adjacent packets of different classes [7], a blank packet is inserted if the packet interval is more than 1s, and the packet within 1s is ignored. The blank packet uses a 256-dimensional stream of all 1s to represent the payload, which can prevent the parameters of each neuron in the neural network from being invalidated by multiplying by 0 when encountering a blank packet.

Finally, the preprocessed packet stream set *P* is obtained, and the model can be input in the next step.

### 3.2. CNN Module

**Figure 2:**
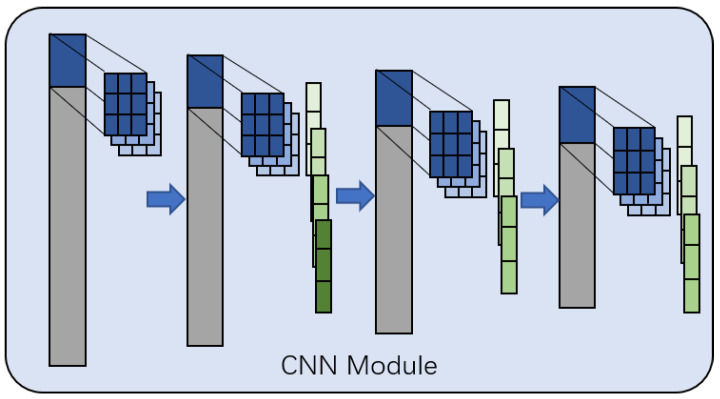
The framework of the CNN module.

The structure of the CNN module is shown in Figure 2. It consists of a 1D-CNN model, including four convolutional layers and three pooling layers.

The input is a 256-dimensional vector p, the kernel size of the first convolutional layer is 3, and the number of output channels is 10; therefore, the output is a 10×254-dimensional matrix. The kernel size of the second convolutional layer is also 10. After the convolutional layer, a max pooling layer with a pooling size of 3 and a stride length of 1 is applied, and the number of output channels is 20; therefore, the output is a 20×250-dimensional matrix. The third and fourth layers are consistent with the second layer method, and the number of output channels is 10 and 1 respectively, resulting to a 242-dimensional vector as output. Finally, a Dense module is connected for dimensionality reduction to facilitate the subsequent operation of the BERT module.

### 3.3. BERT Module

**Figure 3:**
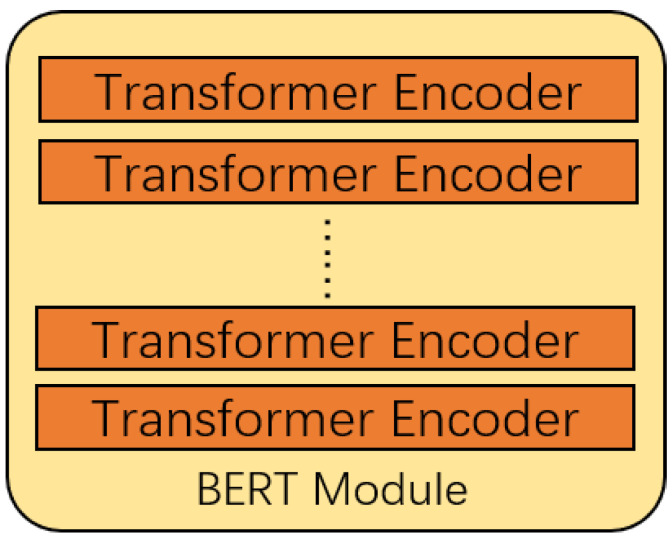
The framework of the BERT module.

The structure of the BERT module is shown in Figure 3, based on the BERT model in [25].

The BERT model [21] is mainly composed of the Encoder of the Transformer model [17]. The encoder has six layers, and each layer contains two sub-layers, which are a multi-head attention mechanism and a fully connected feedforward network. A sentence *X* is input into the Encoder, $X\in R^{batch\text{\_}size\times seq\text{\_}len}$, whose dimension is [batch_size,seq_len].

Initially, position embedding is performed, obtaining [batch_size,seq_len,embed_dim]-dimension $X_{embed}\in R^{batch\text{\_}size\times seq\text{\_}len\times embed\text{\_}dim}$,

(2) $$X_{embed}=EmbeddingLookup\left(X\right)+PositionEncoding.$$

For the multi-head attention mechanism sub-layer, to learn the expression of multiple meanings, a linear mapping is made of $X_{embed}$. Three weights are assigned $W_{Q},W_{K},W_{V}\in R^{embed\text{\_}dim\times embed\text{\_}dim}$, and three matrices Q,K,V are formed after linear mapping, which is consistent with the dimension before linear transformation.

(3) $$Q=Linear\left(X_{embed}\right)=X_{embed}W_{Q},$$

(4) $$K=Linear\left(X_{embed}\right)=X_{embed}W_{K},$$

(5) $$V=Linear\left(X_{embed}\right)=X_{embed}W_{V}.$$

The number of heads is defined as h,head_size=embed_dim/h. After splitting according to head_size, the dimensions of Q,K,V are [batch_size,seq_len,h,embed_dim/h], after transposition is [batch_size,h,seq_len,embed_dim/h].

For the *i*-th head, the dimensions of $Q_{i},K_{i},V_{i}$ are all [batch_size,seq_len,embed_dim/h]; then, the output of the *i*-th head is

(6) $$head_{i}=Attention\left(Q_{i},K_{i},V_{i}\right)=softmax\left(\frac{Q_{i}K_{i}^{T}}{\sqrt{d_{k}}}\right)V_{i}.$$

where $d_{k}$ is the dimension of $K_{i}$, $d_{k}=\left[batch\text{\_}size,seq\text{\_}len,embed\text{\_}dim/h\right]$.

For the multi-head attention mechanism sub-layer, the information of each head is connected to obtain $X_{hidden}:\left[batch\text{\_}size,seq\text{\_}len,embed\text{\_}dim\right]$,

(7) $$X_{hidden}=MultiHead\left(Q,K,V\right)=Concat\left(head_{1},...,head_{h}\right).$$

Then, residual connection and normalization are performed. Since the dimensions of $X_{embed}$ and $X_{hidden}$ are the same, we can directly add the elements to make the residual connection. Then, this is normalized to the standard normal distribution, obtaining $LayerNorm(X_{embed}+X_{hidden})$.

After each sub-layer, a residual connection and normalization will be added, so the output of each sub-layer is

(8) SubLayer_output=LayerNorm(X+(SubLayer(X))).

The BERT model [25] contains two tasks. The first task, Masked Language Model (MLM), is a token-level task that can solve the problem where bidirectional model causes the predicted next word to appear in a given sequence. A part of the token is randomly masked in proportion, so that the model predicts and restores the part that is covered or replaced. The second task, Next Sentence Prediction (NSP), is a sentence-level task. Since many NLP downstream tasks are based on the relationship between sentences, it is necessary to determine whether two adjacent sentences are contextual.

In the BERT module we designed, we set the number of layers of Transformer Encoder to 4, 8 and 12, respectively. Since each flow segment contains 10 consecutive packets, each packet becomes a packet stream after preprocessing. After adding the time interval, a flow segment contains up to 15 packets. Therefore, to enable a flow segment to be input into the BERT module for processing at the same time, it is assumed that a flow contains 15 packets. If there are fewer than 15 packets, blank packets are inserted before completion. That is, the BERT module gathers 15 outputs of the previous module as input at a time. A Dense module is also connected after the BERT module for final classification.

### 3.4. Dense Module

**Figure 4:**
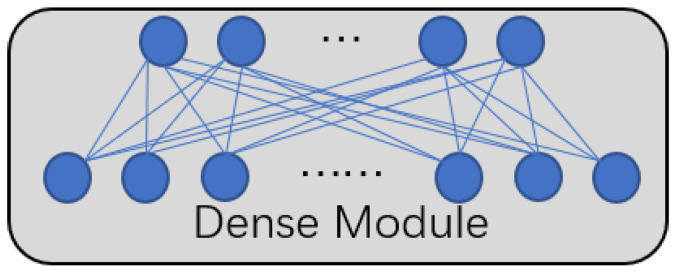
The framework of the Dense module.

The structure of the Dense module is shown in Figure 4, which is mainly composed of a fully connected layer.

After the CNN module and BERT module, there are Dense modules, respectively. Dense module 1 after the CNN module contains a fully connected layer, which can change the dimension of the output of the CNN module to a dimension suitable for the input of the BERT module. Dense module 2 after the BERT module is also a fully connected layer, and the specific parameter settings are determined by the number of final classification classes.

### 3.5. Pre-Training of Unlabeled Data

The pre-training proposed in this paper contains two stages, which correspond to the two tasks of the BERT model pre-training.

The first stage is based on the packet level, which corresponds to the token-based MLM task in the BERT model. This stage mainly trains the model’s understanding of encrypted packets. By calculating the entropy value of the payload, each packet is divided into a plaintext packet and a ciphertext packet.

For a packet, extract the payload part, get $p=\{x_{1},x_{1},\ldots ,x_{n}\}$, calculate the entropy of each packet,

(9) $$H=-\sum_{i=1}^{n}P\left(x_{i}\right)\log_{2}P\left(x_{i}\right),1\leq i\leq n,$$

Entropy is a measure of the degree of chaos in the system. The larger the entropy, the more chaotic the system and the less information it carries. For plaintext payload and ciphertext payload, the principle is similar. The entropy of the plaintext payload should be much smaller than the entropy of the ciphertext payload, because encrypted data have high randomness and do not contain obvious information. By calculating and comparing experimental data, we set the threshold of entropy $H_{0}=4$.

(10) $$\left\{\begin{array}{cc} H\left(p\right)<H_{0}, & p\in P_{plain}\\ H\left(p\right)\geq H_{0}, & p\in P_{cipher}. \end{array}\right.$$

when $H\geq H_{0}$, the packet is considered to be a ciphertext packet; when $H<H_{0}$, the packet is considered to be a plaintext packet.

The labeled plaintext and ciphertext packets are utilized to train the model, to identify encrypted packets. After completing the first stage of pre-training, proceed to the second stage of tasks.

The second stage is based on the flow level, which corresponds to the sentence-based NSP task in the BERT model. This stage mainly trains the model’s understanding of a flow, that is, the understanding of the relationship between packets. Initially, positive and negative sample sets are constructed.

The positive sample set $S^{+}$ contains *n* positive samples,

(11) $$S^{+}=\left\{s_{1}^{+},s_{2}^{+},...,s_{n}^{+}\right\}.$$

the positive sample $s^{+}$ is defined as a continuous flow *F*, where each flow contains 10 consecutive packets, denoted as

(12) $$s_{i}^{+}≜F^{i}=\left\{p_{1}^{i},p_{2}^{i},...,p_{10}^{i}\right\},1\leq i\leq n.$$

The negative sample set $S^{-}$ has the same number as the positive sample set $S^{+}$, and contains *n* negative samples.

(13) $$S^{-}=\left\{s_{1}^{-},s_{2}^{-},...,s_{n}^{-}\right\}.$$

the negative sample $s^{-}$ is defined as a discontinuous flow $\bar{F}$, which is obtained by transforming the positive sample. Each packet in the positive sample is replaced with other packets with a certain probability, and the sample after the replacement is called a negative sample.

(14) $$s_{i}^{-}≜\bar{F^{i}}=\left\{f\left(p_{1}^{i}\right),f\left(p_{2}^{i}\right),...,f\left(p_{10}^{i}\right)\right\},1\leq i\leq n,$$

(15) $$f\left(p_{j}^{i}\right)=\left\{\begin{array}{cc} p_{j}^{i} & \left(P=0.7\right)\\ p_{j^{\prime }}^{i^{\prime }} & \left(P=0.3\right) \end{array}\right.,\left(i^{\prime },j^{\prime }\right)\neq \left(i,j\right),1\leq j\leq 10.$$

The labeled positive and negative sample sets are utilized to train the model so that it can distinguish whether a flow is continuous. After completing the pre-training, the CBD model will be further fine-tuned according to the downstream task—encrypted traffic classification—to achieve the final goal.

### 3.6. Model Transfer and Fine-Tune

In the entire CBD model, some modules will perform the transfer based on supervised learning. In this transfer process, the structure of the module is fixed, but the parameters will change according to the tasks after the transfer. This transfer method is also called parameter fine-tuning under supervised learning in the deep model.

It should be noted that, in addition to the three modules in the fine-tuning phase, we will also transfer the CNN module and the Dense module in the pre-training phase.

After the CBD model is fine-tuned, it will output the predicted classification results in the test phase. We will evaluate the classification results in the next section.

## 4. Experiment

This section mainly describes the experimental settings, experimental evaluation metrics, and specific experimental results to verify the effectiveness of the CBD model proposed in this paper.

### 4.1. Experimental Settings

This paper used the public dataset ISCXVPN2016 [8] published by the Canadian Institute of Cyber Security, University of New Brunswick in the downstream task of encrypted traffic classification. We choosed the traffic data of the two social networks Facebook and Skype as the experimental data. Facebook traffic included two applications: chat and audio, and Skype traffic included two applications, chat and file transfer. The traffic of each specific application can be encapsulated by VPN protocol, or just ordinary network traffic nonVPN. The experiment used eight classes of data, a total of 8000 samples. Each class randomly selects 1000 samples from ISCXVPN2016, and each sample is a flow segment, that is, it contains 10 consecutive packets. The specific data classes are shown in Table 1.

In the pre-training process, the plaintext packet in the first stage is composed of 256-byte plaintext, and the ciphertext packet is composed of randomly selected data, other than the eight classes of data mentioned above, regardless of the class and data volume. In the second stage, 5000 samples are randomly selected to generate a positive sample set, and the class is also ignored. The negative sample set is obtained through the transformation of the positive sample set.

### 4.2. Evaluation Metrics

When evaluating the performance of a model, the class of interest is usually regarded as the positive class, and the other classes are regarded as the negative class. The evaluation index is usually formulated with four basic conditions: True Positive (TP), which predicts the positive class as a positive class. False Positive (FP), predicts the negative class as a positive class. True Negative (TN), predicts the negative class as a negative class. False Negative (FN), predicts the positive class as a negative class. We use five commonly used evaluation metrics—Accuracy, F1-score, Precision, Recall, and Area Under Curve (AUC)—as a basis for evaluating the performance of the model.

Accuracy is the ratio of the number of correctly classified samples to the total number of samples for a given data.

(16) $$Accuracy=\frac{TP+TN}{TP+TN+FP+FN}=\frac{n_{correct}}{n}.$$

where $n_{correct}$ represents the number of samples that are correctly predicted, and *n* represents the total number of samples.

F1-score is the harmonic average of Precision and Recall. Precision refers to the proportion of the real class in the sample predicted as the positive class, and Recall refers to the proportion of all the positive classes that are predicted to be the positive class.

(17) $$\frac{2}{F1\text{-}score}=\frac{1}{Precision}+\frac{1}{Recall},$$

(18) $$F1\text{-}score=2Precision\times \frac{Recall}{Preciaion+Recall},$$

(19) $$Precision=\frac{TP}{TP+FP},$$

(20) $$Recall=\frac{TP}{TP+FN}.$$

In multi-class problems, we calculate the macro-F1-score. The macro-F1-score calculates the F1-score for each class separately, and then takes the unweighted average. Macro-precision and macro-recall are the same.

(21) $$macro\text{-}F1\text{-}score=\frac{1}{C}\sum_{i=1}^{C}F1\text{-}score_{i},$$

(22) $$macro\text{-}Precision=\frac{1}{C}\sum_{i=1}^{C}Precision_{i},$$

(23) $$macro\text{-}Recall=\frac{1}{C}\sum_{i=1}^{C}Recall_{i}.$$

where *C* represents the number of classes.

The ROC Curve (receiver operating characteristic curve) is a curve obtained by using the False Positive Rate (FPR) as the *x*-axis and True Positive Rate (TPR) as the *y*-axis. The larger the area of the AUC, the better the classification effect. FPR represents the probability that negative samples are mistakenly classified as a positive class, and TPR represents the probability of positive samples are correctly classified as a positive class.

(24) $$TPR=\frac{TP}{TP+FN},$$

(25) $$FPR=\frac{FP}{FP+TN}.$$

### 4.3. Experimental Results

The CBD model selects 4-layer, 8-layer, and 12-layer BERT for experiments. The experiment is an eight-class experiment; that is, the random classification accuracy rate is 12.5%. In the experiment, we found that an eight-layer BERT can achieve the best results.

In order to demonstrate the performance of our proposed model, we perform experiments on a small-scale dataset, as shown below:

1. Sample Size = 20% or 0.2 (200 samples are randomly selected from each class of data to form a new dataset);
2. Sample Size = 40% or 0.4 (400 samples are randomly selected from each class of data to form a new dataset);
3. Sample Size = 60% or 0.6 (600 samples are randomly selected from each class of data to form a new dataset);
4. Sample Size = 80% or 0.8 (800 samples are randomly selected from each type of data to form a new dataset).

**Figure 5:**
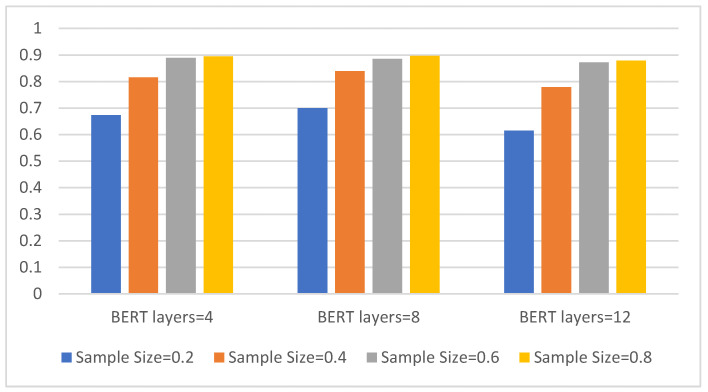
The three models with different BERT layers and eight-class accuracy on four sample sizes. The abscissa is the BERT model with three different layers, the ordinate is the eight-class accuracy, and the four colors represent four different sample sizes.

Additionally, we perform eight-class experiments on four different sample sizes to compare three types of BERT. The results are shown in Figure 5.

It can be seen from Figure 5 that the classification accuracy of all three models increases as the sample size increases. Overall, the eight-layer BERT model has the best effect. It can achieve a classification accuracy of 70% in a sample size of 0.2, and it also performs best in a sample size of 0.4, reaching a classification accuracy of approx. 84%. As the sample size increases, the advantages of the eight-layer BERT model compared to the other two are no longer obvious.

Table 2 shows the detailed comparison results under the four metrics.

It can be seen from Table 2 that, when the number of layers is too low, the model learning ability is poor, the improvement speed is slow, and the final performance is not high enough. When the number of layers is too high, the model is too complex, and when the sample size provided by the target task is limited, the model’s improvement performance is limited. Therefore, in the experiment, the performance of the CBD model of the eight-layer BERT is the best.

**Figure 6:**
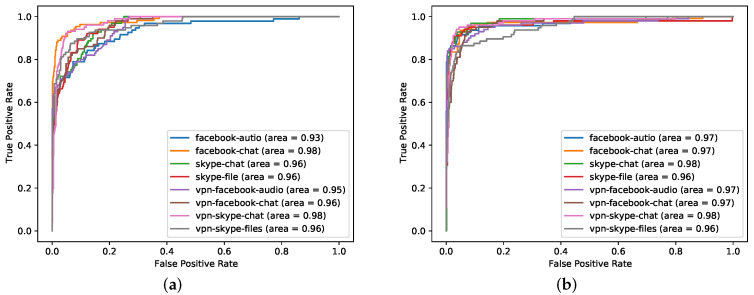
ROC curve of eight-layer BERT (best) at 40% sample size. The picture on the left (**a**) is the model without pre-training, and the picture on the right (**b**) is the model with pre-training. The abscissa is the FPR, and the ordinate is the TPR. The eight colors represent the eight classes of data in the downstream tasks.

The general pre-training method is an important part of the CBD model. To verify the effectiveness of the pre-training method, we compare the CBD model with the no pre-training CBD model, and the results are shown in Figure 6.

It can be seen from Figure 6 that no pre-training model performed worse than original CBD model. For a complex model with many BERT layers and many neuron parameters, the accuracy without pre-training is not good. This is due to insufficient data and insufficient model learning. However the pre-trained model has been fully learned, and the higher the number of layers layers, the stronger the learning ability. The model has learned the hidden features in the data, so the accuracy is high. However, after reaching a certain level, the performance improvement brought by increasing the number of layers is very limited. In addition, we found that, without pre-training, the smaller the sample size, the more the number of training failures.

In order to verify whether the number of pre-training epochs has an effect on the final classification result, we set different pre-training epochs and compared the experimental results. The results are shown in Table 3.

**Figure 7:**
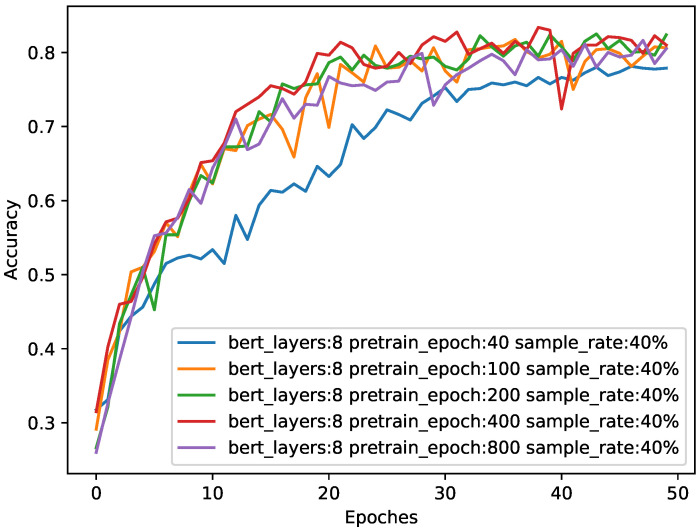
The impact of different pre-training epochs on accuracy when sample size is 0.4. The abscissa is the number of epochs during training in the downstream task, and the ordinate is the classification accuracy of the test. The four colors represent the performance of the eight-layer BERT in different epochs during pre-training.

We also observed the changes in the model accuracy during the training process, and the results are shown in Figure 7.

It can be seen from Table 3 and Figure 7 that the higher the number of pre-training epochs, the more adequate the pre-training, and the better the performance of the model. However, after more than 200 epochs, the increase in pre-training brings little performance improvement. Therefore, we chose the optimal pre-training epochs number of 200.

**Figure 8:**
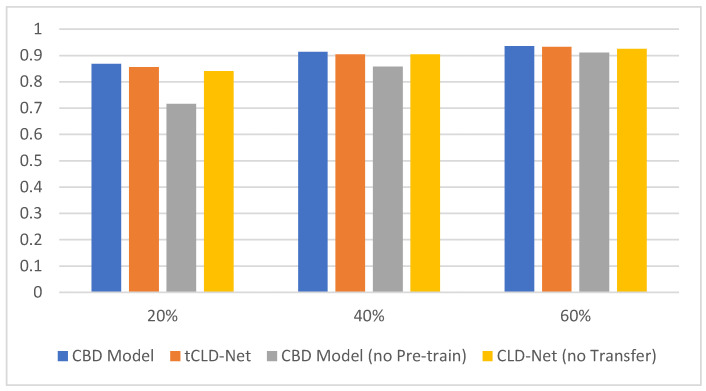
Test results on the three-class target domain. The abscissa represents the sample size, and the ordinate represents the accuracy of classification. The four colors represent CBD Model, tCLD-Net, CBD Model without pre-training and CLD-Net without transfer.

To verify the indispensability of each module in the CBD model, we compare the CBD model with other models. First, the necessity of the BERT module is verified. tCLD-Net is similar to the CBD model, which combines deep learning and transfer learning. The difference is that tCLD-Net did not use the BERT module, but the LSTM module. We compared the two models in the same dataset. The dataset is in the target domain mentioned in [14]. The comparison results are shown in Figure 8.

It can be seen from Figure 8 that the CBD Model performs better than tCLD-Net on the three-class dataset. However, it is worth noting that the pre-training of tCLD-Net (also called source domain training) is based on label data, and a large amount of artificially labeled label data need to be provided. For the CBD Model, the performance is similar when the target task training set is the same, and the pre-training data do not need to be artificially provided with labels. In addition, the performance of the no pre-training CBD Model is worse than the no pre-training CLD-Net when the amount of data is insufficient. This is caused by the high complexity of the model and insufficient amount of data.

In order to verify the necessity of CNN module, we removed the CNN module in the CBD model. An embedding layer was directly embedded on the ciphertext packet, and then the BERT module and the Dense module were connected to form an Embedding BERT Dense (EBD) model. The results of a comparison between the performance of the EBD model and the CBD model are shown in Table 4.

It can be seen from Table 4 that the classification effect of the EBD model is poor, and the time efficiency of the EBD model in the experiment is very low. When BERT is directly used for data with a close contextual correlation, the effect is better, but the context correlation of encrypted data is deliberately confused and the correlation is weak; therefore, the effect of the EBD model is poor. The reason for the unsatisfactory effect of the EBD model may be that a sample in the experiment is 10 consecutive packets randomly selected from a flow, and the sample data are likely to be ciphertext packets. If the experiment does not randomly select, but selects the first 10 packets of a flow as a sample, it is likely to obtain plaintext packets or handshake packets. At this time, the performance of the EBD model may be better than the current results. Therefore, CNN has an irreplaceable role.

In addition, if there is no CNN module and no Embedding layer, directly using the Bert module to learn the packet payload is equivalent to encoding the payload, and every two bytes are mapped to a number, that is, 0-65535. In this encoding process, every two bytes correspond to a word in NLP. At this time, when this model is iterated to 60 epochs, the classification accuracy of 8-class fluctuates at around 10%, and the highest is 10.25%. Therefore, every module in the CBD model is indispensable.

**Figure 9:**
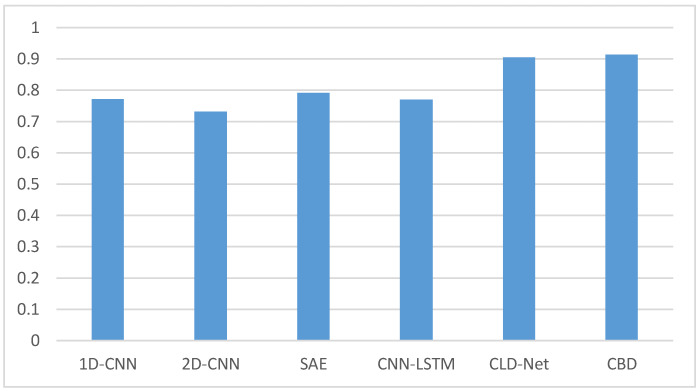
Accuracy comparison results of several models when the sample size is 0.4.

We also select five models in the existing researches to compare with the CBD model. The five models are one-dimensional CNN (1D-CNN) and two-dimensional CNN (2D-CNN) mentioned in [5], Stacked Autoencoder (SAE) mentioned in [2], a combination of CNN and LSTM (CNN-LSTM) mentioned in [4], and CLD-Net mentioned in [7]. The five models are all performed on the dataset mentioned in this paper. The comparison results are shown in Figure 9.

The comparative experiments are implemented on a three-class dataset. It can be seen from Figure 9 that the accuracy of the CBD model is the highest, reaching more than 91%. CLD-Net also has good results, which may be related to the traffic recombination strategy mentioned in [4]. The accuracy of 1D-CNN, 2D-CNN, SAE, and CNN-LSTM models are all less than 80%. This may be due to the small sample size and the simple structure of several models, which makes it unable to learn and understand the hidden features of encrypted traffic. At the same time, the sample of our experiment is a random 10 consecutive packets without header information or handshake phase packets. Some existing traffic classification models may not randomly select packets in their own experiments. This contains not only the payload, but also other information. However, the data in the real situation often only have payload information, so the effect of experimenting with several existing models is not ideal.

## 5. Conclusions

In this paper, we proposed an encrypted traffic classification model with a general pre-training method. Compared with other traffic classification methods that combine deep learning and transfer learning, this model can directly learn the basic characteristics of traffic data from unlabeled data, and use CNN and BERT to automaticly learn the features of traffic from the perspective of packets and flows. The experiment was performed on the public dataset, and a class-balanced dataset was constructed for eight-class tasks. When the sample size was only 0.4 and the number of BERT layers was eight, the CBD model could achieve a classification accuracy of more than 91% in three-class tasks. In eight-class tasks, the classification accuracy rate could reach about 84%, which was nearly 8% higher than that of the model without pre-training. The eight-class tasks refer to the encrypted traffic classification tasks performed on the balanced dataset proposed in this paper, and the three-class tasks refer to the classification tasks performed on the target domain dataset mentioned in [14].

In future work, we will consider the classification of unknown classes of encrypted traffic to further fit the actual network environment. At the same time, the classification of real-time network traffic will also be regarded as the research goal of the next stage. Another research direction is designing a model that uses less time and computational resource, which can detect and classify unknown traffic, and supports real-time updates. 

## Figures and Tables

**Table 1:** Data classes of encrypted traffic.

Encrypted Application Traffic Classes	VPN	Facebook	vpn-facebook-chat
			vpn-facebook-audio
		Skype	vpn-skype-chat
			vpn-skype-file
	nonVPN	Facebook	facebook-chat
			facebook-audio
		Skype	skype-chat
			skype-file

**Table 2:** Classification performance of models with different BERT layers—sample size=0.4, pre-training epoch = 200.

	Models	4-Layer BERT	6-Layer BERT	8-Layer BERT	10-Layer BERT	12-Layer BERT
Metrics						
Accuracy		0.8163	0.82	**0.8388**	0.8263	0.7788
macro-F1-score		0.8147	0.8197	**0.8397**	0.8242	0.7793
macro-Precision		0.8161	0.8225	**0.8449**	0.8321	0.7924
macro-Recall		0.8150	0.8192	**0.8381**	0.8239	0.7769

**Table 3:** The classification performance of models with different pre-training epochs when the number of BERT layers is eight and sample size is 0.4.

	Epochs	40	100	200	400	800
Metrics						
Accuracy		0.8062	0.8175	**0.8388**	0.8338	0.8163
macro-F1-score		0.8042	0.8160	**0.8397**	0.8341	0.8146
macro-Precision		0.8127	0.8184	**0.8449**	0.8370	0.8170
macro-Recall		0.8053	0.8161	**0.8381**	0.8335	0.8149

**Table 4:** Evaluation metrics of CBD model and EBD model when BERT layers = 8, sample size = 0.4, and pre-training epoch = 200.

	Models	CBD	EBD
Metrics			
Accuracy		**0.8388**	0.5462
macro-F1-score		**0.8397**	0.5372
macro-Precision		**0.8449**	0.5693
macro-Recall		**0.8381**	0.5512

## Data Availability

Publicly available datasets were analyzed in this study. This data can be found here: https://www.unb.ca/cic/datasets/vpn.html (accessed on 18 June 2021).
